# Parietal alpha and theta power predict cognitive training gains in middle-aged adults

**DOI:** 10.3389/fnagi.2025.1530147

**Published:** 2025-03-13

**Authors:** Luka Juras, Ivana Hromatko, Andrea Vranic

**Affiliations:** Department of Psychology, Faculty of Humanities and Social Sciences, University of Zagreb, Zagreb, Croatia

**Keywords:** predictors, working memory, cognitive training, EEG power, middle-age adults

## Abstract

Research on executive functions training shows inconsistent outcomes, with factors like age, baseline cognitive abilities, and personality traits implicated as predictive of training gains, while limited attention has been given to neurophysiological markers. Theta and alpha band power are linked to cognitive performance, suggesting a potential area for further study. This study aimed to determine whether relative theta and alpha power and their ratio could predict gains in updating and inhibition training beyond the practice effects (the order of training session). Forty healthy middle-aged adults (aged 49–65) were randomly assigned to either the cognitive training group (*n* = 20), or the communication skills (control) group (*n* = 20). Both groups completed the self-administered training sessions twice a week for 10 weeks, totaling to 20 sessions. Resting-state EEG data were recorded before the first session. Mixed-effects model analyses revealed that higher relative parietal alpha power positively predicted training performance, while theta power negatively predicted performance. Additionally, higher parietal alpha/theta ratio was associated with better training outcomes, while the frontal alpha/theta ratio did not demonstrate significant predictive value. Other EEG measures did not show additional predictive power beyond what was accounted for by the session effects. The findings imply that individuals with specific EEG pattern may change with cognitive training, making resting-state EEG a useful tool in tailoring interventions.

## Introduction

Cognitive training, a standardized repeated practice in cognitive tasks targeting distinct abilities, with the goal of enhancing overall cognitive and everyday functioning (Nguyen et al., 2019), has recently garnered significant attention. The presumed benefits of cognitive training protocols have been studied across various age groups, but the public interest is predominantly focused on alleviation of age-related symptoms of cognitive decline. Impairments of cognitive functioning affect up to one third of older adults (Birle et al., 2021). Due to the growing share of older adults worldwide, even mild cognitive impairment (MCI), an intermediary stage between normal aging and dementia, is becoming a pressing public health issue (Petersen et al., 2017). Cognitive aging processes tax executive functions (EF), including updating, inhibition, and cognitive flexibility, which are crucial for daily functioning and overall cognitive health (Heckner et al., 2021). Consequently, many cognitive training programs are aimed specifically at enhancing EF. Despite this growing interest, findings on the efficacy of EF training programs remain inconsistent, with varied outcomes among participants (Ali et al., 2022; Nguyen et al., 2019; Soveri et al., 2017). While studies often focused on demographic and psychological factors, the exploration of neurophysiological markers as predictors of training gains remains relatively underexplored.

### The relevance of resting state EEG for cognitive training research

Electroencephalography (EEG) has long been recognized as a valuable non-invasive tool for diagnosing cognitive impairments, and identifying abnormalities indicative of neurodevelopmental and neurological disorders, sometimes even before pathological symptoms are manifested (Meghdadi et al., 2021; Putman, 2011; Canuet et al., 2011). Resting-state EEG measures brain activity when an individual is at rest, revealing rhythmic patterns that offer valuable insights into neurophysiological underpinnings of cognitive processes (Buzsáki, 2006; Niedermeyer and Lopes da Silva, 1999). Some features of resting state EEG correlate with normal aging (Chen et al., 2023; Finnigan and Robertson, 2011; Caplan et al., 2015; Knyazeva et al., 2018), while others reveal specific patterns in older adults with cognitive issues, including an increase in low-frequency (delta, theta) and a decrease in high-frequency (alpha, beta) power (Buzi et al., 2023; Moretti et al., 2011; Babiloni et al., 2013; Miranda et al., 2019). Such changes in the resting-state EEG frequencies may reveal broader trends in cognitive efficiency, marking them as promising markers of cognitive health or decline (Choi et al., 2024, Perez et al., 2024), which might carry the potential for preemptive interventions.

There has been an abundance of research confirming the link between EEG features and cognitive functioning in healthy populations. Findings suggesting human EEG as a time stable trait of a feasible genetic base (Kondacs and Szabó, 1999; Smit et al., 2005) provide additional support for the notion that inter-individual variations in quantitative measures of EEG represent valid neurophysiological markers of cognitive functioning. EEG features have been linked to numerous aspects of cognitive functioning, including academic achievement (Cheung et al., 2014), problem solving (Kounios et al., 2006), processing speed (Klimesch et al., 1996) and intelligence (Thatcher et al., 2005; Doppelmayr et al., 2005; Jaušovec, 2000).

Pertaining to the EF training protocol, the focus of our study is aligned with the EEG indicators of EF. Earlier studies found a positive correlation between both, resting state alpha peak frequency (Clark et al., 2004) and resting state theta power (Finnigan and Robertson, 2011), and working memory (WM) performance. Ambrosini and Vallesi (2016), found a relation between resting state EEG alpha and beta wave asymmetry and task-switching. This asymmetry may reflect transient and sustained cognitive control abilities, which are crucial for adaptive behavior and flexibility in task-switching. Basharpoor et al. (2021) found that coherence of alpha, beta, and theta bands between left and right frontal regions, as well as the coherence of beta and theta bands in the left frontal regions predict EF. However, Gordon et al. (2018) used a comprehensive set of EF tasks in healthy adult sample and found no meaningful relation between spectral power measures and WM, task-switching, and inhibition. More central to the aim of our study, are findings of interventional studies, especially those showing that cognitive training changes oscillatory activity in alpha and theta bands (Langer et al., 2013; Spironelli and Borella, 2021). Similarly, research on fronto-parietal transcranial alternating current stimulation (tACS) demonstrates that targeted EEG modulation can enhance WM, particularly by increasing the coherence in fronto-parietal networks (Pupíková et al., 2024). This suggests that shifts in EEG properties might be a reflection of the effects cognitive training has on specific neural networks. The growing body of research illustrates EEG’s potential not only to assess, but also to modulate cognitive function, paving the way for personalized, EEG-guided therapeutic strategies.

### Resting state EEG markers as indicators of cognitive reserve

If we consider cognitive reserve (Stern et al., 2019) as the brain’s resilience to age-related cognitive decline and neurodegenerative diseases, which enables the maintenance of cognitive functioning despite brain aging or pathology, the above-mentioned EEG markers could be considered its correlates. They are both associated with various cognitive processes pertaining to the concept of cognitive reserve and show specific changes in mature and older populations. Excessive theta power in older adults has been linked to attentional lapses and slower cognitive processing, which are more common in individuals with lower cognitive reserve (Katayama et al., 2024). EEG research on individuals with MCI and dementia has consistently identified characteristic neural changes, marked by an increase in delta and theta, alongside a decrease in alpha and beta activity (Prichep et al., 2006; Musaeus et al., 2018). Moreover, some studies have reported associations between EEG power and biomarkers, such as tau protein (Smailovic et al., 2018). Finnigan and Robertson (2011) argued that two forms of theta-frequency oscillations may exist; one indicative of healthy neurocognitive function and the other, EEG/alpha slowing linked to future substantial cognitive decline. Thus, cognitive resilience might be mediated by both, the relative theta power and the degree of alpha slowing. Similarly, Cummins and Finnigan (2007) found that lower theta power was associated with healthy cognitive aging, indicating that high cognitive reserve in older adults is often marked by the reduced (both resting and task-related) state theta.

Considering the above-mentioned findings in both healthy and cognitively impaired populations, we opted to further explore the notion that EEG markers could provide insights into the mechanisms underlying cognitive training gains. More specifically, the present study aims to investigate the role of theta and alpha power as predictors of EF training gains among middle-aged adults. Cognitive aging involves dynamic changes throughout the lifespan. Although a decline in fluid abilities is generally observed beginning in the early 50 s (e.g., Glahn et al., 2013), middle-aged adults remain relatively underrepresented in cognitive training research. In this population, decline in cognitive abilities is subtle, suggesting that interventions at this stage may be especially beneficial in building up the cognitive reserve well into older age. We hypothesized that, above the effects of training sessions, both alpha and theta activity shall predict training gains, albeit in different directions. By addressing these questions, this study seeks to contribute to a deeper understanding of the neurophysiological mechanisms underlying cognitive training and to inform the development of more effective, individualized training interventions.

## Methods

### Participants

A sample of 40 adults, aged 47–65, participated in the study. Participants were randomly assigned to either the Updating training (UT; *n* = 20) group or the Communication skills training (CT; *n* = ~ 20) group, i.e., control group. There was no significant age difference between the two groups (M_*UT*_ = 54.9, SD_*UT*_ = 3.82; M_*CT*_ = 55.7, SD_*CT*_ = 4.01; *t* (38) = −0.65, *p* = 0.523). Both groups consisted predominantly of female participants (13 in the UT group, 14 in the CT group) and had similar educational backgrounds, with seven participants in UT and six in CT holding a degree lower than a BA (X^2^ (3) = 3.27, *p* = 0.351). Participants reported no severe visual or hearing impairments, psychiatric or neurological disorders, anti-dementia medication use, or other work-related limitations.

### Procedure

Participants were informed that the study aimed to investigate the effects of various activities on cognitive abilities. Participants completed the criterion N-back task before first and after the last session. EEG data were collected during a resting state (eyes open for 2 min) before the first training session and during the initial UT session. Testing and EEG recordings were conducted in non-shielded environments, either at the participants’ homes or workplaces.

The study was approved by the Ethical Committee of the research institution, and all participants provided a written informed consent in accordance with the Declaration of Helsinki.

### Outcome measure

#### N-back task

The *n*-back task (Jaeggi et al., 2010) is a widely used measure of memory updating. In this task, participants are presented with a sequence of stimuli for 500 ms, followed by a 2500 ms interstimulus interval. They are instructed to press a specific key whenever the current stimulus matches the one presented *n* trials earlier. The stimuli consisted of eight photographs from the Karolinska Directed Emotional Faces (KDEF) database (Lundqvist et al., 1998), featuring a male or a female model displaying one of four basic emotions: sadness, happiness, anger, or surprise. The task included three difficulty levels: 1-back, 2-back, and 3-back. After completing a practice round, participants progressed through a block of 20+ n stimuli for each difficulty level, starting with 1-back, and with each level repeated twice. The score is calculated as the proportion of hits minus the proportion of false alarms across a session (Snodgrass and Corwin, 1988).

#### Training

Both groups (UT and CT) completed two self-administered training sessions per week for a period of 10 weeks, totaling to 20 sessions. Each session lasted approximately 20 min and was accessed on participants’ personal computers. Performance was monitored throughout the training, with performance data recorded for later analysis.

#### N-back training

The UT group engaged in an adaptive version of the *n*-back task. Training began with the 1-back condition, and the difficulty of subsequent blocks was adjusted based on participants’ performance. If a participant made fewer than two errors in a block, they advanced to the next level (n + 1). Conversely, if they made five or more errors, the next block was set to a lower level (n–1). Points were awarded for each completed block, corresponding to the difficulty level. At the end of each session, participants earned virtual medals based on the highest level reached: bronze for 2-back, silver for 3-back, and gold for 4-back or higher. In subsequent sessions, participants started one level below the highest level achieved in the previous session. Each training session consisted of 15 blocks, with each block containing 20 + n stimuli.

#### Communication skills training

The CT group participated in a computer-based communication skills training program, adapted from Jaušovec and Jaušovec (2012). This CT included 20 interactive online presentations, covering both the theoretical foundations of communication processes and practical strategies for improving communication efficiency. At the end of each session, participants completed a quiz. Feedback was provided in the form of points, which were awarded based on a predefined scoring system.

#### EEG data recording and preprocessing

EEG data were recorded using a Mobita 32-Channel Wireless EEG System (Biopac Systems Inc.), with electrodes placed according to the international 10/20 extended system: Fp1, Fpz, Fp2, F7, F3, Fz, F4, F8, FC5, FC1, FC2, FC6, T7, C3, Cz, C4, T8, TP9, CP5, CP1, CP2, CP6, TP10, P7, P3, Pz, P4, P8, PO, O1, Oz, and O2. EEG was recorded during a 2-min resting state with eyes open and throughout the first UT session. For the analysis, electrodes were grouped by region: frontal electrodes included Fp1, Fp2, F7, F3, Fz, F4, and F8, while parietal electrodes included Pz, P3, P4, P7, P8, and PoZ.

EEG data from both conditions were preprocessed and analyzed using MATLAB (Version R2020b) and the FieldTrip toolbox (Oostenveld et al., 2011). Preprocessing began with filtering the data using a high-pass filter at 1 Hz and a low-pass filter at 40 Hz. The signals were re-referenced to the average of all electrodes. Data were then segmented into non-overlapping 2-s epochs for both the resting state and task performance periods. Visual inspection and artifact rejection were performed to eliminate noisy segments. Independent Component Analysis (ICA) was applied to remove artifacts related to eye movements, followed by a second visual inspection to ensure clean data.

The power spectrum was computed using a multi-taper Fast Fourier Transform (FFT) with a frequency resolution of 0.5 Hz. Relative power was calculated as the ratio of power in a specific frequency band to the total power from 1 to 30 Hz. The alpha band was defined as 8–12 Hz and the theta band as 4–8 Hz. Relative power was calculated since it takes into account individual differences in skull thickness and volume conduction.

### Data analysis

Statistical analyses were performed using R software (version 4.0.2; R Core Team, 2023) with the nlme package (Pinheiro et al., 2024). To examine whether training led to improved performance on the trained n-back task, we conducted a 2 (training and control) × 2 mixed-design (pretest and posttest) ANOVA.

To examine whether theta and alpha power predicted n-back task performance during training, we applied multilevel modeling. The dependent variable was the average session score. We compared multiple models: (1) a null model, (2) linear time as a predictor, (3) quadratic time change, and (4) models incorporating alpha and theta power across frontal and parietal sites. To systematically assess these combinations, we used a topographical approach, entering frequency bands from the same location and time point (resting state and first training session) into a single model. One model included frontal alpha and theta, another parietal alpha and theta, and so on. For the alpha/theta ratio, both frequencies were combined into a single marker, allowing the inclusion of frontal and parietal sites. We used the maximum likelihood (ML) method and compared models via chi-square tests (*p* < 0.05). All predictors, including time, were mean-centered before analysis to enhance interpretability.

## Results

**FIGURE 1:**
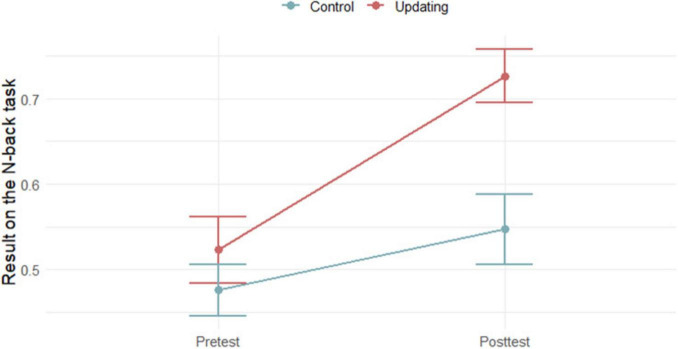
Results of participants in the criterion *n*-back task at the pretest and the posttest in Updating training group and control group (*N* = 40).

**FIGURE 2:**
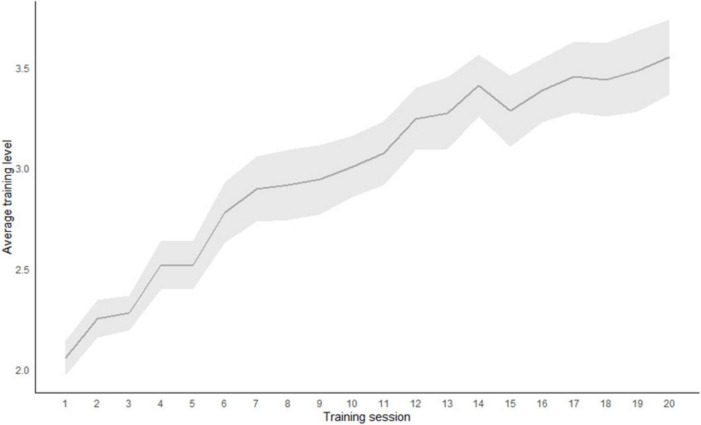
Average training score across 20 training sessions in the Updating training group (*N* = 20).

Results in the criterion *n*-back task at both pretest and posttest in training and control group are presented in Figure 1. Based on the two-tailed *t*-tests, no initial differences were found on criterion task at the pretest (*p* = 0.950). Mixed design 2 × 2 ANOVA showed a significant main effect of the measurement point (*F* (1,38) = ~ 20.35; *p* < 0.001; η^2^ = 0140) and a main effect of the group (*F* (1,38) = 7.84; *p* = 0.008; η^2^ = 0.097). The interaction of the measurement point and the group is also statistically significant (*F* (1,38) = 4.78; *p* = 0.035; η^2^ = 0.033). Only participants in the *n*-back UT group improved in their performance. Average training performance across 20 training sessions is shown in Figure 2.

Given that the mixed design ANOVA indicated that participants in the UT group exhibited greater performance improvements than those in the CT group, it is essential to explore the individual predictors of training performance within the UT group. To this end, we conducted a mixed-effects model (MLM) analysis aimed at examining how specific EEG markers predict training performance (average difficulty of each training session) among participants in the UT group (see Table 1). The intraclass correlation coefficient (ICC) indicated that approximately 52.6% of the total variance could be attributed to differences between participants, justifying the use of this analysis.

**TABLE 1:** Linear and quadratic changes in the average number of points in the Updating training group (*n*_1_ = 400 observations, *n*_2_ = 20 participants).

	Model 0	Model 1	Model 2	Model 3	Model 4	Model 5	Model 6	Model 7	Model 8
**Fixed effects**									
Intercept	2.98** (0.14)	3.00** (0.13)	3.10** (0.14)	3.10** (0.12)	3.10* (0.13)	3.1** (0.11)	3.1** (0.13)	3.1** (0.12)	3.1** (0.13)
Session		0.08** (0.003)	0.08** (0.003)	0.08** (0.003)	0.08** (0.003)	0.08** (0.03)	0.08** (0.03)	0.08** (0.03)	0.08** (0.03)
Session^2^			−0.003** (0.001)	−0.004** (0.001)	−0.004** (0.001)	−0.004** (0.001)	−0.003** (0.001)	−0.003** (0.001)	−0.003** (0.001)
R0 frontal alpha				2.47 (1.30)					
R0 frontal theta				−3.68 (2.42)					
T1 frontal alpha					−1.81 (1.86)				
T1 frontal theta					−2.42 (2.36)				
R0 parietal alpha						1.75* (0.8)			
R0 parietal theta						−6.57** (2.26)			
T1 parietal alpha							0.04 (1.77)		
T1 parietal theta							−0.02 (2.67)		
R0 frontal alpha/theta								−0.22 (0.27)	
R0 parietal alpha/theta								0.39* (0.18)	
T1 frontal alpha/theta									−0.28 (0.11)
T1 parietal alpha/theta									0.15 (0.36)
**Random effects**									
σ_e_	0.35	0.13	0.12	0.11	0.11	0.11	0.11	0.11	0.11
σ_0_	0.32	0.36	0.37	0.29	0.31	0.22	0.34	0.26	0.34
Deviance statistic	740.9	384.2	350.4	346.9	348.5	341.5	353.3	344.5	351.1
Model (df): X^2^		0–1 (1): 356.4**	1–2 (1): 34.5**	2–3 (2): 4.1	2–4 (2): 2.3	2–5 (2): 9.3*	2–6 (2) 0.1	2–7 (2): 6.0*	2–8 (2): 0.3

Legend: R0 – resting-state; T1 – first training session. Model 0 – Null model; Model 1 – Time as a linear predictor; Model 2 – Model 1 + quadratic time as a predictor; Model 3 – Model 2 + resting-state eyes-open relative frontal alpha and theta power; Model 4 – Model 2 + relative frontal alpha and theta power during the first training session; Model 5 – Model 2 + resting-state eyes-open relative parietal alpha and theta power; Model 6 – Model 2 + relative parietal alpha and theta power during the first training session; Model 7 – Model 2 + resting-state eyes-open frontal and parietal alpha/theta power ratio; Model 8 – Model 2 + Parietal alpha/theta power ratio during the first training session.

*p < 0.05;

**p < 0.01.

The results indicated that training performance could be explained by both linear and quadratic functions (Model 2), suggesting that participants improved their performance across sessions, with greater improvements observed in the earlier sessions and a slowing of this improvement in later sessions. We compared Model 2, which included only training sessions as predictors, with models that incorporated EEG markers as predictors of training performance. To mitigate potential multicollinearity, we analyzed six models, each containing two predictors.

The findings revealed that higher relative parietal alpha power was a significant positive predictor of training performance, while theta power was a significant negative predictor (Model 5). Additionally, a higher parietal alpha/theta ratio was associated with better training outcomes; however, the frontal alpha/theta ratio did not demonstrate significant predictive value (Model 7). Furthermore, other predictors did not contribute additional predictive power beyond what was accounted for by the session effects.

## Discussion

Several observations stem from this study. Firstly, our participants improved their performance across sessions, implying that middle aged populations do benefit from these types of interventions, at least within the trained task limits. However, it should also be noted that greater improvements were observed in earlier training sessions, followed by a slowing of this improvement in later sessions, implying that in order to keep participants’ intrinsic motivation, and still obtain similar results, training protocols – at least for middle aged and older populations - might be shortened. We opted for the 20 sessions protocols, as suggested by the meta-analyses (Lampit et al., 2014), but the dropout rate (7 participants, as compared to 3 in the control group), as well as the qualitative feedback received by our participants, suggest that most of them struggled to keep up with the training.

Regarding our main aim, the findings revealed that higher relative parietal alpha power positively predicted training performance, while theta power negatively predicted performance. Additionally, a higher parietal alpha/theta ratio was associated with better training outcomes, while the frontal alpha/theta ratio did not demonstrate significant predictive value. This is in line with earlier studies showing that increased alpha activity in parietal regions is associated with attentional control and working memory in various populations. Studies have shown that alpha power in these regions is linked to attentional focus and cognitive efficiency, which supports better performance in various cognitive tasks (Klimesch, 1999; Nunez and Srinivasan, 2006). The positive relationship between higher parietal alpha power and cognitive training performance in our study also suggests that participants with a more pronounced alpha activity in this region were better equipped to benefit from training. This could mean that individuals who can achieve or maintain higher parietal alpha power might have a cognitive advantage during training.

The negative association between theta power and training performance suggests that elevated theta activity, particularly in the parietal region, may be less conducive to successful training outcomes. Again, this finding is in line with others, suggesting that elevated theta power can indicate a less optimal cognitive state for certain tasks. While task-related theta power shows positive associations with cognitive performance, resting theta power tends to have negative correlations with cognitive performance (Tan et al., 2024). This inverse relation between resting and task-related powers within specific bands of EEG spectra has been observed in several earlier studies: within alpha frequency band, high resting state power appears to be associated with a large amount of desynchronization during task performance, while the opposite is true for theta, i.e., low resting power predicts large synchronization or power gains during task performance (Doppelmayr et al., 1998; Vogt et al., 1998; Klimesch et al., 2000).

While theta is associated with memory and learning, high levels of theta are also related to drowsiness or cognitive fatigue, especially in older adults, and there is a growing evidence suggesting that higher resting theta is associated with lower EF in older children and adolescents (Tan et al., 2024). While moderate theta activity may support memory functions, excessive theta can be indicative of impaired cognitive control or vigilance, impacting task performance in aging adults. Elevated theta power has been implicated in attentional lapses and cognitive slowing, which may reduce effectiveness in cognitively demanding tasks.

In aging populations, heightened theta power may reflect age-related cognitive decline or difficulties in sustaining attention and processing of new information, factors which could interfere with the learning process. Thus, lower theta power might serve as an indicator of better baseline cognitive function or resilience (Prichep et al., 2006; Musaeus et al., 2018), enabling more effective engagement in training. The significant role of the parietal alpha/theta ratio as a predictor of training gain underscores the balance between alpha and theta activity as a potential marker of cognitive efficiency. It has been shown that the relation between alpha/theta ratio and cognitive performance is age-dependent (Trammell et al., 2017) and our finding suggests that among middle-aged participants a higher alpha/theta ratio reflects an efficient cognitive state, potentially indicating a brain that is less prone to distractibility, thus allowing for greater cognitive engagement. Klimesch (1999) emphasized the importance of the alpha/theta ratio as an indicator of brain efficiency, particularly in relation to memory and attentional control. It seems that in cognitive training interventions, this ratio could be used as a biomarker for predicting or even enhancing training responsiveness in elderly individuals.

The finding that the frontal alpha/theta ratio did not predict performance suggests a region-specific effect, with parietal rather than frontal brain areas playing a more central role in training-related cognitive gains. It is widely accepted that EF are mainly regulated by the frontal lobes (Miyake et al., 2000; Miller and Cohen, 2001); however, some non-frontal brain regions including the parietal cortex, as well as some sub-cortical structures, such as the basal-ganglia and the cerebellum are also heavily involved (Alvarez and Emory, 2006; Friedman and Miyake, 2017). The lack of explanatory power of frontal activity could also indicate that in this context cognitive training gains rely more on processes related to attentional orientation and sensory integration. This implies that training approaches tailored to engage and monitor parietal rather than frontal activation might yield better outcomes. Such a capitalization of the potential for cognitive plasticity in aging midlife individuals suggests that the benefits of training may rely as much on the sustained engagement as on the baseline neurophysiological profiles.

Our study has several limitations. While the sample size meets the recommended 20 participants per group (Simons et al., 2016), it remains relatively small, potentially limiting the detection of smaller effects. Additionally, using a convenience sample with highly educated participants reduces the generalizability of findings. The home-based training setting provided less control over conditions compared to a laboratory, where training effects are often stronger (Schwaighofer et al., 2015). Another limitation is our focus on predicting performance during training rather than examining transfer effects. Therefore, it is important to note that the same task used as the outcome measure was also employed during the training.

### Potential applications

Obviously, the training process (practice, repetition, and learning engagement) is critical to cognitive gains. The finding that certain parameters of resting state EEG can predict training gains beyond the practice effect adds to the current knowledge regarding the predictors of training outcomes and potentially open a new venue toward individualized cognitive trainings. There are several ways in which these findings might be incorporated when designing personalized trainings. For example, if participant’s initial resting state alpha power is low, an intervention aimed at boosting alpha power generally (such as relaxation exercises or mindfulness practices) or alpha/theta ratio specifically (via neurofeedback) might be applied before the cognitive training sessions. One might also monitor theta power to gauge cognitive engagement vs. fatigue. Since high theta power may indicate cognitive fatigue or difficulty in sustaining attention, for participants with elevated theta, it may be beneficial to reduce task complexity or session length. This structure can help maintain attention and prevent cognitive overload, potentially leading to better training outcomes.

## Conclusion

In summary, our findings suggest that relative parietal alpha, theta and their ratio, could serve as useful indicators for cognitive training responsiveness in mid-aged adults. The insights into theta power’s negative role further refines our understanding of EEG biomarkers, highlighting the importance of maintaining an optimal balance of brain activity for effective cognitive processing. The results also suggest that cognitive training interventions may benefit from a focus on parietal rather than frontal brain activity, perhaps through targeted neurofeedback or cognitive training protocols that reinforce parietal activity. These findings contribute to our understanding of the brain’s adaptability in aging populations and can be informative for the design of tailored cognitive training programs that leverage EEG biomarkers for more personalized and effective interventions.

## Data Availability

The raw data supporting the conclusions of this article will be made available by the authors, without undue reservation.
